# Effects of Dual-Release Hydrocortisone on Bone Metabolism in Primary and Secondary Adrenal Insufficiency: A 6-Year Study

**DOI:** 10.1210/jendso/bvad151

**Published:** 2023-12-06

**Authors:** Valeria Hasenmajer, Davide Ferrari, Dario De Alcubierre, Valentina Sada, Giulia Puliani, Ilaria Bonaventura, Marianna Minnetti, Alessandra Tomaselli, Riccardo Pofi, Emilia Sbardella, Alessia Cozzolino, Daniele Gianfrilli, Andrea M Isidori

**Affiliations:** Department of Experimental Medicine, “Sapienza” University of Rome, Rome 00161, Italy; Department of Experimental Medicine, “Sapienza” University of Rome, Rome 00161, Italy; Department of Experimental Medicine, “Sapienza” University of Rome, Rome 00161, Italy; Inserm U1052, CNRS UMR5286, Claude Bernard Lyon 1 University, Cancer Research Center of Lyon, Lyon 69373 CEDEX 08, France; Department of Experimental Medicine, “Sapienza” University of Rome, Rome 00161, Italy; Oncological Endocrinology Unit, IRCCS Regina Elena National Cancer Institute, Rome 00128, Italy; Department of Experimental Medicine, “Sapienza” University of Rome, Rome 00161, Italy; Department of Experimental Medicine, “Sapienza” University of Rome, Rome 00161, Italy; Department of Experimental Medicine, “Sapienza” University of Rome, Rome 00161, Italy; Oxford Centre for Diabetes, Endocrinology and Metabolism, NIHR Oxford Biomedical Research Centre, University of Oxford, Churchill Hospital, Oxford OX3 7LE, UK; Department of Experimental Medicine, “Sapienza” University of Rome, Rome 00161, Italy; Department of Experimental Medicine, “Sapienza” University of Rome, Rome 00161, Italy; Department of Experimental Medicine, “Sapienza” University of Rome, Rome 00161, Italy; Department of Experimental Medicine, “Sapienza” University of Rome, Rome 00161, Italy; Centre for Rare Diseases (Endo-ERN accredited), Policlinico Umberto I, Rome 00161, Italy

**Keywords:** dual-release hydrocortisone, adrenal insufficiency, osteoporosis, glucocorticoids, bone metabolism, trabecular bone score

## Abstract

**Context:**

Patients with primary (PAI) and secondary adrenal insufficiency (SAI) experience bone metabolism alterations, possibly due to excessive replacement. Dual-release hydrocortisone (DR-HC) has shown promising effects on several parameters, but bone metabolism has seldom been investigated.

**Objective:**

We evaluated the long-term effects of once-daily DR-HC on bone in PAI and SAI.

**Methods:**

Patients on immediate-release glucocorticoid therapy were evaluated before and up to 6 years (range, 4-6) after switching to equivalent doses of DR-HC, yielding data on bone turnover markers, femoral and lumbar spine bone mineral density (BMD), and trabecular bone score (TBS).

**Results:**

Thirty-two patients (19 PAI, 18 female), median age 52 years (39.4-60.7), were included. At baseline, osteopenia was observed in 38% of patients and osteoporosis in 9%, while TBS was at least partially degraded in 41.4%. Higher body surface area–adjusted glucocorticoid doses predicted worse neck (*P* < .001) and total hip BMD (*P* < .001). Longitudinal analysis showed no significant change in BMD. TBS showed a trend toward decrease (*P* = .090). Bone markers were stable, albeit osteocalcin levels significantly varied. PAI and SAI subgroups behaved similarly, as did patients switching from hydrocortisone or cortisone acetate. Compared with men, women exhibited worse decline in TBS (*P* = .017) and a similar trend for neck BMD (*P* = .053).

**Conclusion:**

After 6 years of chronic DR-HC replacement, BMD and bone markers remained stable. TBS decline is more likely due to an age-related derangement of bone microarchitecture rather than a glucocorticoid effect. Our data confirm the safety of DR-HC replacement on bone health in both PAI and SAI patients.

Adrenal insufficiency (AI), either primary (PAI) due to adrenal failure or secondary (SAI) due to impaired adrenocorticotropic hormone (ACTH) secretion by the pituitary gland, is characterized by inappropriately low endogenous glucocorticoid (GC) [1] levels and requires long-term GC replacement therapy. Conventional GC replacement regimens include short-acting oral hydrocortisone and cortisone tablets, which require multiple daily administrations or synthetic steroid use. These fail to mimic the physiological circadian rhythm of cortisol secretion, exposing the patient to multiple peaks and troughs [2, 3].

Studies assessing bone mineral density (BMD) in AI patients receiving chronic GC replacement therapy have yielded controversial results [4], with values varying from normal [5-7] to decreased [8-12] in most published series. Collectively, an increased risk of osteoporotic fractures has been described in AI [11-15], and recent reports have suggested that patients with PAI might exhibit an increased rate of hip [14] and vertebral fractures [13] despite normal bone density, suggesting the presence of skeletal fragility irrespective of BMD values [13, 16, 17]. Trabecular bone score (TBS), an indirect indicator of bone microarchitecture, has emerged as a predictor of osteoporotic fracture risk independently of BMD [18, 19]. Nevertheless, TBS remains poorly characterized in patients with AI, with only one study reporting similar values between 29 patients with PAI and a matched control group [20]. PAI and SAI are also very different regarding concomitant comorbidities and gender differences.

A potential correlation between bone mineral loss and the overall daily dosage of GCs has been suggested [5, 6, 8, 13, 21-23]. However, epidemiological studies have not been yet conclusive in linking the daily dosage to the complications of the disease [24-27], instead suggesting a selective, dose-dependent adverse impact of more potent, long-acting formulations [5, 7, 28]. Of note, chronic hydrocortisone usage has not been associated with an increased risk of fractures within normally administered doses [28].

Disruption of the physiological circadian profile of cortisol secretion has been identified among the mechanisms underlying the many comorbidities observed in AI patients, including osteoporosis and metabolic derangement [29, 30]. Two modified-release hydrocortisone tablets have entered the market as new formulations that better mimic cortisol secretion's circadian rhythm [31]. The once-daily dual-release hydrocortisone (DR-HC) is the first to become available, and it has been proven superior to the conventional multiple-daily-dosing therapy to improve metabolic and immune alterations in patients with AI [32-34].

Recent longitudinal studies have suggested that DR-HC might benefit bone health, reporting a significant increase in lumbar and femur neck BMD in patients with PAI [35] and SAI [36] following the switch from conventional GC treatment to DR-HC. However, PAI and SAI patients have never been evaluated in the same study for a sufficiently long period, and the effects of DR-HC on bone microarchitecture have never been compared between the 2 etiologies.

Our study aims to characterize bone metabolism in a cohort of AI patients and evaluate the effects of DR-HC replacement therapy over a long-term follow-up.

## Materials and Methods

### Study Design and Participants

This is a real-life, longitudinal observational study. Patients with AI referring to our center were shifted to DR-HC in the context of a randomized controlled trial [32] (NCT02277587) at baseline (unbiased patient selection). After study termination, all enrolled patients on DR-HC continued their therapy and underwent bone health evaluations according to clinical practice. Patients with PAI or SAI aged 18 to 80 years were evaluated at baseline while on long-term, stable, immediate-release GC therapy and then at 24, 48, and 72 months after switching to DR-HC. In accordance with current recommendations [29], patients on immediate-release hydrocortisone and cortisone acetate were shifted to an equivalent daily dose of DR-HC, administered once daily in the morning. Dietary calcium intake was assessed at each study time point. Patients not reaching the age-appropriate required intake were supplemented with calcium carbonate or calcium citrate.

Exclusion criteria for the present study were concomitant or previous acromegaly and Cushing syndrome, pregnancy or breastfeeding, untreated hypogonadism and GH deficiency, early menopause, previous high dosage GC treatment, untreated or uncontrolled diabetes mellitus (DM), malabsorption syndromes, any clinically relevant kidney, respiratory, hepatic disease, malignancies, and chemotherapy during the previous 5 years before enrollment. In addition, patients were excluded if they exhibited one or more of the following: onset of menopause at least 1 year before enrollment or throughout the study timeframe, anabolic or antiresorptive therapy for osteoporosis, hyperparathyroidism, hypoparathyroidism, or any other causes of secondary osteoporosis [37].

The original study was approved by the Local Ethics Committee of Sapienza University in Rome and was conducted in accordance with the Declaration of Helsinki (1964) and its subsequent amendments. All enrolled patients provided their written informed consent to participate in the study.

### Procedures

Throughout the study timeframe, patients underwent the following assessments.

#### Biochemical panel

At each time point, 24-hour urine samples were collected, and blood samples were drawn after overnight fasting between 8:00 and 10:00 Am, 2 to 3 hours after taking the daily morning GC dose. The biochemical evaluations included: hormonal panel, including ACTH, renin, serum cortisol, urinary free cortisol (UFC); bone metabolism assessment, including calcium, phosphate, magnesium, 25OH-vitamin D (vitamin D), alkaline phosphatase (ALP), osteocalcin (OCN), C-terminal telopeptide (CTX), parathormone (parathyroid hormone; PTH). Serum and urine analyses were all performed at a central laboratory with standard analytic procedures.

#### Dual-energy x-ray absorptiometry assessment

Bone densitometry studies were performed at the study center at each time point by a member of the study medical staff with expertise in dual-energy x-ray absorptiometry (DXA) analysis. BMD was assessed at lumbar spine, femur neck, and total hip (Hologic APEX software version 4.5.3). The Least Significant Changes were 0.010 g/cm^2^ for femur neck and total hip BMD and 0.008 g/cm^2^ for lumbar BMD. According to the Position Statement of the International Society for Clinical Densitometry (ISCD Official Positions—Adult; https://www.iscd.org/official-positions/2019), in postmenopausal women and male patients aged 50 years or older, BMD was expressed as T-score, comparing results with those of sex-matched Caucasian population at peak of bone mass. A T-score equal to or less than −2.5 SD was defined as osteoporosis, while a T-score between −1.0 SD and −2.5 SD was defined as low bone mass or osteopenia. In patients younger than 50 years old, the results were expressed as Z-scores comparing the results with those of age and sex-matched Caucasian population, and a Z-score equal to or less than −2.0 SD was defined as “below the expected range for age.” BMD was also reported in absolute values (g/cm^2^) for all sites mentioned above.

Bone microarchitecture was analyzed using the TBS iNsight software (v. 2.1.2.0) by evaluating pixel gray-level variations in the corresponding lumbar spine DXA scan. Following the results of a meta-analysis [38], the TBS was used to categorize bone microarchitecture as either normal (TBS > 1.310), partially degraded (TBS between 1.230 and 1.310), or degraded (TBS < 1.230).

Vertebral imaging (x-ray) was available at baseline in postmenopausal women or in men aged >50 years, and after 5 years in absence of clinical signs for incident fractures. Other fragility fractures were evaluated as reported by patients.

### Statistical Analysis

The normality of distribution was assessed by the Shapiro-Wilk test. Continuous variables were reported as mean ± SD when normally distributed or as median and interquartile range (IQR, 25%-75%) otherwise. Comparison of variables between groups was assessed by independent samples *t* test or Mann-Whitney test, according to data distribution. The difference between the binomial proportions of 2 independent groups on a dichotomous dependent variable has been assessed by the chi-square test for homogeneity. Pearson's correlation coefficient estimated correlations between normally distributed continuous variables.

Linear and logistic regression analyses were performed, as appropriate, to identify predictors of bone parameters at baseline and at the subsequent time points of the study. Differences in BMD, TBS, and bone marker levels between different time points and baseline were reported as mean percentage of change [%change_1-2_ = (T_2_−T_1_)*100/T_1_] or mean difference [ΔT_1-2_ = T_2_−T_1_]. To assess any significant intrapatient changes between time points, repeated measure analysis using a mixed-effects model with the Restricted Maximum Likelihood (REML) estimation for random effects was used. This approach allowed us to account for within-subject correlations while managing the missing data. One-way analysis of covariance (ANCOVA) was performed to evaluate any differences in BMD and TBS values variation between PAI and SAI patients while controlling for age and baseline BMD. Bonferroni correction for multiple comparisons was applied. A *P* value of less than 0.05 was regarded as significant. All statistical analyses were performed using SPSS for Windows, version 26 (SPSS, Inc.) and GraphPad Prism version 9.

## Results

### Baseline Characteristics

According to the strict eligibility criteria, 32 participants (19 PAI; 13 SAI) of the original DREAM trial cohort could enter the current study. The baseline characteristics of the study population are available in Table 1. Among them, 18 patients (56.3%) were female, 9 of whom (50.0%) were postmenopausal, and 14 patients were male, 9 of whom (64.3%) were older than 50 years. The median age was 52.0 years (39.4–60.7), and the median disease duration was 41 months (24–96). Before switching to DR-HC, GC treatment consisted of twice- or thrice-daily short-acting hydrocortisone in 19 patients (59.4%) and twice-daily cortisone acetate in 13 patients (40.6%). The median body surface area (BSA)-adjusted baseline daily GC dose was 13.2 mg/m^2^ hydrocortisone equivalent.

**Table 1.** Baseline characteristics

	Whole cohort (N = 32)	PAI patients (N = 19)	SAI patients (N = 13)	*P* value
General characteristics				
Age, years	52.0 (39.4–60.7)	46.6 (36.2–57.8)	52.8 (51.1–61.3)	.192
Sex, M/F	14/18	7/12	7/6	.341
Disease duration, months	41 (24–96)	60 (32–240)	24 (23–36)	**.009**
Menopause, n (% of women)	9 (50%)	4 (33.3%)	5 (83.3%)	.**046**
Autoimmune comorbidities	11 (34.4)	11 (57.9%)	0	.**001**
Other endocrine deficiencies				
GHD	1 (3.1)	0	1 (7.7)	.219
Hypothyroidism	15 (46.9)	8 (42.1%)	7 (53.8)	.513
Hypogonadism, n (% of males)	5 (35.7)	1 (5.3%)	4 (30.8)	.051
Baseline GC replacement therapy				
Hydrocortisone	19 (59.4)	11 (57.9)	8 (61.5)	.837
Cortisone acetate	13 (40.6)	8 (42.1)	5 (38.5)	.837
Baseline BSA-adjusted HC equivalent daily dose, mg/m^2^	13.2 (10.3–16.2)	14.7 (12.2–17.0)	11.0 (10.2–12.9)	.**050**
Baseline BSA-adjusted DR-HC daily dose, mg/m^2^	12.8 (11.1–14.7)	14.1 (12.7–15.4)	11.0 (10.2–12.7)	.**001**
Bone turnover markers				
Vitamin D, ng/mL	33.6 (13.1)	36.9 (14.8)	26.2 (1.6)	.**019**
ALP, U/L	63.4 (15.8)	59.1 (14.2)	70.4 (16.5)	.090
PTH, pg/mL	35.0 (26.3–55.3)	31 (25.8–58.9)	36.8 (31–50)	.918
OCN, ng/mL	24.8 (21.9–28.2)	25.7 (24.4–29.4)	21.9 (15.6–24.6)	.033
CTX, ng/mL	0.54 (0.28)	0.64 (0.23)	0.36 (0.26)	**.041**
Bone health evaluation				
Osteoporosis*^a^*	3 (9.4)	1 (5.3)	2 (15.4)	.335
Low bone density*^a^*	12 (37.5)	5 (26.3)	7 (53.8)	.114
Bone density below the expected range for age*^a^*, n*^b^*	1 (7.1)	1 (9.1)	0	.588
TBS, mean (SD)	1.332 (0.101)	1.364 (0.095)	1.280 (0.090)	.024
Normal TBS	17 (58.6)	12 (66.7)	5 (45.5)	.260
Partially degraded TBS	8 (27.6)	6 (33.7)	2 (18.2)	.376
Degraded TBS	4 (13.8)	0	4 (36.4)	**.006**

Data are expressed as mean (SD) or median (interquartile range [IQR], 25%-75%) for continuous variables and as n (%) for categorical variables.

Abbreviations: ALP, alkaline phosphatase; BSA, body surface area; CTX, C-terminal telopeptide; DR-HC, dual-release hydrocortisone; GC, glucocorticoid; GHD, growth hormone deficiency; HC, hydrocortisone, OCN, osteocalcin; PAI, primary adrenal insufficiency, PTH, parathyroid hormone; SAI, secondary adrenal insufficiency, TBS, trabecular bone score.

^*a*^At one or more analyzed sites (total hip, femur neck, lumbar spine).

^*b*^(% of 14 patients evaluated with Z-score).

Regarding endocrine status, 15 patients (46.9%) were on thyroid replacement therapy, 5 male patients (35.7%) were on testosterone replacement for hypogonadism, 3 patients (9.4%) had type 2 DM, 1 patient (3.1%) had type 1 DM, and 1 patient (3.1%) had growth hormone deficiency. Seventeen PAI patients (89.5%) were on fludrocortisone treatment at a median dose of 0.05 mg/day (0.05–0.1). All hormone deficiencies were on long-term, stable replacement therapy from at least 1 year before baseline and were adequately replaced throughout the study timeframe.

At baseline, 12 patients had low BMD, 1 patient (40 years of age) had bone density below the expected range for age, and 3 patients had osteoporosis; 58.6% of patients had normal TBS, 27.6% had partially degraded TBS, and 13.8% had degraded TBS. All patients had normal calcium and phosphate levels. Patients meeting the guideline criteria [39] were already on regular cholecalciferol supplementation at baseline, with mean serum vitamin D levels in the whole cohort being 33.6 ± 13.1 ng/dL. Levels of ALP, CTX, vitamin D, PTH, and OCN were not different between patients with normal and decreased BMD, while OCN levels were lower in patients with altered (partially degraded/degraded) TBS (26.8 ng/mL [23.9–34.8] vs 22.3 ng/mL [19.9–24.3], *P* = .043)

In the whole cohort, total daily BSA-adjusted GC dose effectively predicted femur neck (B = −.017, *P* < .001) and total hip BMD (B = −.018, *P* < .001). Similarly, UFC levels significantly predicted lumbar BMD (B = −.001, *P* = .024). Disease duration showed no associations with BMD, TBS, or serum bone markers.

#### Baseline comparison between PAI and SAI patients

A comparison of baseline characteristics between PAI and SAI patients is summarized in Table 1.

In our cohort, SAI patients were generally older than PAI patients, although the age difference did not reach statistical significance (PAI 46.6 years [36.2–57.8] vs SAI 52.8 years [51.1–61.3], *P* = .192). The prevalence of menopause was higher in the SAI cohort (33.3% vs 83.3%, *P* = .046) and, as expected, 57.9% of PAI patients showed at least one autoimmune comorbidity, the most frequent one being autoimmune thyroiditis (42.1%). Patients with PAI were on higher daily BSA-adjusted GC dose hydrocortisone equivalent compared to the SAI group (14.7 mg/m^2^ [12.2–17.0] vs 11.0 mg/m^2^ [10.2–12.9], *P* = .050).

Compared to patients with PAI, patients with SAI showed lower vitamin D (36.9 ± 14.8 ng/mL vs 26.2 ± 1.6 ng/mL, *P* = .019), OCN (25.7 ng/mL [24.4–29.4] vs 21.9 ng/mL [15.6–24.6], *P* = .033) and CTX (0.64 ± 0.23 ng/mL vs 0.36 ± 0.26 ng/mL, *P* = .041) levels. In terms of bone microarchitecture, TBS was higher in PAI than SAI (1.364 ± 0.095 vs 1.280 ± 0.090, *P* = .024), and the latter group presented with a higher prevalence of degraded TBS (0% vs 36.4%, *P* = .006), although these results could be age-related and not entirely due to AI etiology.

### Longitudinal Evaluation

Patients were evaluated at baseline and 24, 48, and 72 months after switching to once-daily DR-HC. Data from different time points were allocated to the nearest milestone. Data were available from 19 patients at 24 months, 18 at 48, and 16 at 72 months. Globally, 25% of patients completed all the study time points, while 16% underwent only 3 time points of evaluations (4 at baseline, 24, and 48 months; 3 at baseline, 24, and 72 months; and 1 at baseline, 48, and 72 months). All patients had at least one follow-up time point, and of those who only completed 2 study assessments, 3 were studied at baseline and after 24 months, 9 at baseline and after 48 months, and 4 baseline and after 72 months. The mean follow-up duration of the entire cohort was 57.4 ± 26.4 months.

**Figure 1.**
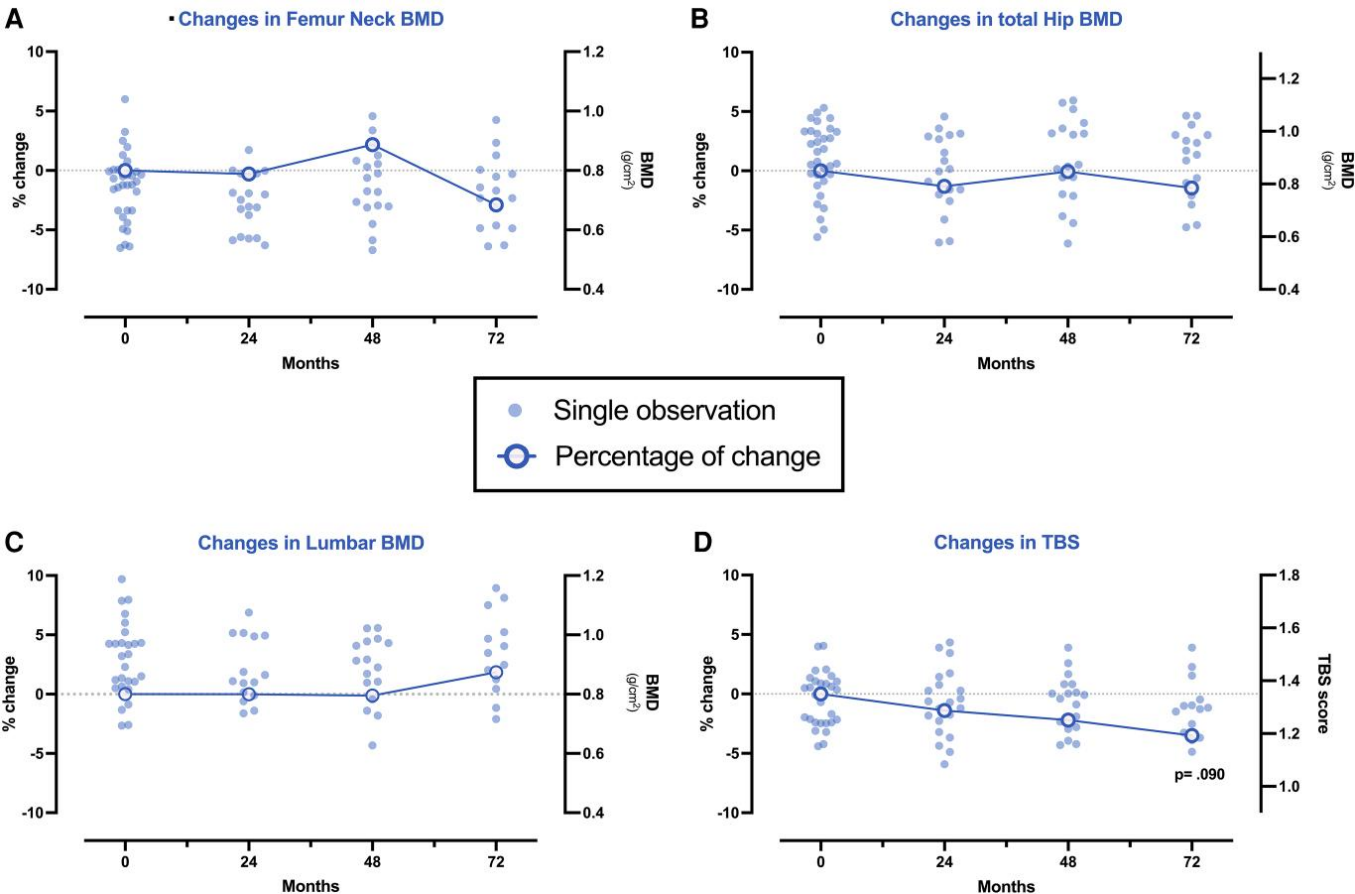
BMD and TBS variations over time in the whole AI cohort: single observations at 24, 48, and 72 months and the respective global percentage changes compared to baseline are reported for BMD at femur neck (A), total hip (B), and lumbar spine (C), and for TBS (D). Abbreviations: TBS, trabecular bone score; BMD, bone mineral density.

The main findings concerning the longitudinal evaluation of BMD, TBS, and biochemical parameters are summarized in Table 2 and Fig. 1. Notably, no significant change from baseline was observed in BMD throughout the longitudinal evaluation at any of the investigated sites (lumbar spine, total hip, femur neck). Results excluding patients with DM and SAI patients with other hormone deficiencies were consistent with the whole cohort (data not shown).

**Table 2.** Longitudinal evaluation in the whole AI cohort

	24 months (n = 19)		48 months (n = 18)		72 months (n = 16)	
	Mean change (SD)	*p^a^* (0–24)	Mean change (SD)	*p^a^* (0–48)	Mean change (SD)	*p^a^* (0–72)
Bone mineral density						
Femur neck	−0.3% (6.7)	1.000	+ 2.2% (7.3)	1.000	−2.9% (6.9)	1.000
Total hip	−1.0% (3.7)	1.000	−0.1% (5.9)	1.000	−1.5% (5.9)	1.000
Lumbar spine, L1-L4	−0.0% (8.6)	1.000	−0.1% (11.5)	1.000	+ 1.8% (13.0)	.972
Trabecular bone score						
TBS, mean (SD)	−1.4% (5.8)	.516	−2.2% (4.8)	1.000	−3.5% (5.8)	.090
Bone turnover markers						
ALP, U/L	−2.3 (9.6)	1.000	+ 5.3 (9.0)	1.000	+2.3 (9.6)	1.000
PTH, pg/mL	−1.8 (12.1)	1.000	−3.7 (11.5)	1.000	−3.2 (20.8)	1.000
CTX, ng/mL	−0.045 (0.167)	1.000	−0.014 (0.174)	1.000	+ 0.006 (0.129)	1.000
OCN, ng/mL	−4.0 (4.2)	.**005**	+ 15.7 (4.0)	.**039**	−0.9 (10.1)	1.000

Evaluation of BMD, TBS, and serum bone markers at 24, 48, and 72 months, considering the whole cohort of AI patients. Data are reported as mean % change (SD) from baseline evaluation for BMD and TBS and as mean difference (SD) for serum bone markers.

Abbreviations: ALP, alkaline phosphatase; CTX, C-terminal telopeptide; OCN, osteocalcin; PAI, primary adrenal insufficiency, PTH, parathyroid hormone; TBS, trabecular bone score.

^*a*^Bonferroni adjusted.

TBS score showed slight variations across the 4 time points (F = 3.623, *P* = .022). Indeed, even though TBS remained stable at 24 and 48 months, a trend toward decrease was observed at 72 months (Δ0–72 = −0.054; 95% CI −0.113 to 0.005; *P* = .090). However, the significance of TBS variations was not maintained after excluding patients with DM (*F* 2.845, *P* = .056) or SAI patients with other hormone deficiencies (*F* = 2.607, *P* = .110). The biochemical assessment (Table 2) showed an overall stability of ALP, PTH, CTX, vitamin D, calcium, and phosphate levels throughout the study. In contrast, OCN levels showed significant changes throughout the study longitudinal observation (*F* = 15.187, *P* = .001): an initial slight decrease was observed at 24 months (Δ0–24 = −6.1 ng/mL; 95% CI −10.4 to −1.8; *P* = .005) followed by an increase at 48 months (Δ24–48 = 19.4 ng/mL; 95% CI 6.5–32.2; *P* = .006), and a subsequent decrease at the 72-month time point (Δ48–72 = −15.8 ng/mL; 95% CI −29.9 to 1.67; *P* = .025). Globally, no significant difference was found in OCN levels at the end of the study compared to baseline (Δ0–72 = −2.5 ng/mL; 95% CI −13.8 to 8.7; *P* = .876).

At the end of the study, 3 patients with osteopenia developed osteoporosis, 2 at the femur neck and 1 at the lumbar spine (Δ_T-score_ = −0.6 SD). No patient with normal bone status developed osteopenia or osteoporosis. TBS status changed from normal to partially degraded in 4 patients (Δ_TBS_ = −0.098) and to degraded in 1 patient (Δ_TBS_ = −0.143), while 2 patients who had partially degraded TBS at baseline developed degraded TBS at 72 months (Δ_TBS_ = −0.094). No patient included in the study had vertebral fractures at baseline, and no vertebral other fragility fractures occurred during the study timeframe.

### Subgroup Analysis

To assess potential differences in bone parameters according to AI etiology, we stratified our cohort, comparing patients with PAI and SAI. The results of this subgroup analysis are detailed in Supplementary Table S1 [40]. At the longitudinal assessment, PAI and SAI did not show any intra-group difference in BMD and TBS across all time points. Similarly, after correcting for age and baseline values, no differences were observed at any time point regarding the rate of change in BMD levels or TBS between PAI and SAI patients (Supplementary Table S1 [40]). These results were confirmed even after excluding patients with type 2 DM. Moreover, no differences were found between PAI and SAI regarding serum bone marker variations at any time point (data not shown).

A sex-stratified analysis was also performed (Supplementary Table S2 [40]). No significant intra- or intergroup differences were observed at 24 and 48 months. However, data from the 72 months time point showed a decrease in TBS (Δ0–72= −0.750; 95% CI −0.143 to −0.006; *P* = .026) in the female group (*F* = 4.224, *P* = .019), even after excluding DM patients. After correcting for age and baseline values, the impairment of TBS at 72 months was confirmed to be higher in women (mean estimated difference −11.2% [−19.6 to −2.9], *P* = .017) compared to men, while a trend toward higher reduction in femur neck BMD was observed (mean estimated difference −7.3% [−14.7 to +0.1], *P* = .053). The higher decline in TBS at 72 months was confirmed after excluding DM patients and SAI patients with other hormone deficiencies (mean estimated difference −9.8% [−19.1 to −0.4], *P* = .045).

Lastly, we investigated the potential relationship between different GC formulations at baseline on bone parameters. Mixed effect analysis revealed no difference within the hydrocortisone and cortisone acetate groups regarding BMD and TBS values across the study time points. Similarly, bone outcomes did not significantly diverge between cortisone acetate and hydrocortisone groups.

## Discussion

The interplay between GCs and bone is well known [41]. In fact, GCs act on all bone cell lineages and can also affect skeletal metabolism via indirect effects, including reduction in insulin-like growth factor 1 (IGF-1) levels, intestinal calcium absorption and relative increase in PTH levels [42].

However, data on GCs and bone health are often obtained in patients with rheumatological or auto-inflammatory disease receiving higher doses of steroid therapy compared to AI patients who often develop glucocorticoid-induced osteoporosis (GIO) [41], and in whom bone impairment is presumably due to different mechanisms. In fact, physiological levels of GC seem to exert a conservative effect on osteoblast function [43], and the role of GC therapy as a replacement for lack of endogenous secretion, rather than for its anti-inflammatory or immune-modulating effects, would suggest little to no impact on bone metabolism in these patients.

Recent data on cardiovascular, immune, and glucose alterations in AI suggest a suboptimal and ultimately not physiological profiles of conventional GC replacement, and the observed increased risk for vertebral fractures and osteoporosis seems to expand this concept to bone health as well [11, 13, 14]. AI patients on GC replacement have a significantly increased risk for vertebral and nonvertebral fragility fractures [11, 13] compared to the general population, with SAI patients showing the highest rates, possibly due to the lack of adequate treatment for concomitant pituitary deficiencies [11]. Conversely, patients with congenital adrenal hyperplasia seem to be more protected, possibly due to the effects of androgens.

Despite a general agreement on impaired bone health in AI patients, the results from studies on bone metabolism in AI are heterogeneous [5, 6, 22, 23]

Our study aimed to provide a well-rounded prospective evaluation of bone health in a selected cohort of patients with PAI and SAI with limited confounding factors, at baseline and after long-term DR-HC therapy. Within our cohort, patients had baseline BMD comparable with published data in the general population [44], while values of TBS were averagely lower than expected [45], with close to 40% of the entire cohort showing at least partially damaged TBS. Although recent studies have confirmed the reliability of BMD in predicting the risk of fractures [46], TBS has proven effective as well in fracture risk assessment [47], especially in endocrine diseases [48] and in GIO [47-49]. However, the observed decline in TBS at 72 months could be explained by growing evidence of a physiological decline in bone microarchitecture with age [6, 50-52]. Further studies in AI will be required to investigate underlying causes and expected trajectories in this condition. The only other available study on TBS values in AI reported comparable results between patients with PAI and sex-matched controls [20]. However, in the work from Zdrojowy-Wełna and colleagues, controls were slightly older than patients, with the age difference almost reaching statistical significance, and mean TBS values were compatible with diagnosis of partially degraded TBS in patients and controls, as reported by the authors. Moreover, in the control group none of the menopausal women were on estrogen replacement, while in the PAI group 40% of menopausal patients were on hormonal replacement. All in all, the lack of observed difference might indicate that bone microarchitecture in PAI patients is in fact more damaged than in the general population, anticipating the detrimental effects of hypoestrogenism and age.

Regarding bone density, trials with various dosages and formulations have been produced in the last decades, with earlier studies hypothesizing, albeit inconsistently, a possible reduction in BMD in PAI patients on stable GC treatment. However, most of those studies were limited by small sample sizes, and the results could be biased by an overall overreplacement typical of the earlier studies [5, 6, 22, 23].

Conversely, our findings appear compatible with those reported by studies on more physiological dosages. A large prospective, cross-sectional study conducted with 81 PAI and 41 CAH patients on physiological replacement therapy (mean hydrocortisone equivalent/day: 12.0 and 15.5 mg/m^2^, respectively), showed BMD values within the reference range of the general population [5]. Similarly, a small study by Chikada et al analyzed bone mineral density via DXA in 10 patients with Addison disease and 5 with isolated ACTH deficiency on physiological GC replacement therapy (hydrocortisone equivalent/day doses ranging from 8.8 to 15.4 mg/m^2^) and reported decreased BMD only in 2 subjects receiving daily hydrocortisone doses of 14.8 and 15.4 mg/m^2^/day [6]. These observations mirror those reported by Zelissen et al, who described normal BMD in male patients with Addison disease treated with hydrocortisone doses of less than 13.6 mg/m^2^/day for at least 10 years [53].

Interestingly, in our study, daily GC dose was inversely correlated with BMD, especially at the femur sites, and UFC levels predicted lumbar BMD impairment. The direct relationship between GC exposure and skeletal damage in AI patients has been explored in several studies [7, 22, 23, 54], pointing toward detrimental effects on BMD mostly related to overreplacement and GC potency. A recent meta-analysis has found a weak correlation between GC dosage and fracture rates in PAI and SAI, but the results did not reach statistical significance, even though the authors suggested that the heterogeneity in age, diagnosis, and criteria for fracture assessment could impair the generalizability of the analyses [11].

Moreover, patients treated with prednisolone have shown worse femur and lumbar Z-scores, a decrease in bone turnover markers and a greater prevalence of osteoporosis compared to those treated with hydrocortisone, suggesting a selective adverse impact of high-potency GC formulations on skeletal health [5, 7]. In our study, however, all patients were on physiological doses of short-acting treatment, and no differences in bone parameters were detected between those on hydrocortisone or cortisone therapy.

Collectively, increased exposure to exogenous GCs, either through higher daily doses or altered metabolism, seems to play a critical role in the deterioration of bone health in individuals with AI. However, while in GIO cumulative exposure to GCs is a relevant aspect for risk of fracture and bone loss [55], in our cohort, bone parameters did not correlate with the disease duration and global exposure. This finding could indicate that, in AI, the correlation with higher daily doses of hydrocortisone equivalent could be due to a greater amplitude of peaks and troughs of circulating steroids and disruption of circadian rhythm rather than to a supra-physiological overall dosage.

Despite the extensive literature focusing on the effect of GC therapy on bone mineralization, the actual correlation between risk of fractures and BMD in patients with AI is still a matter of debate [11, 13, 16]. In our cohort, no incident fractures occurred throughout the study timeframe, but this result could be influenced by the strict inclusion criteria.

DR-HC has shown beneficial effects on immune function, anthropometric measures, and glucose metabolism compared to conventional immediate-release formulations, suggesting a more reliable replication of the physiological cortisol daily curve, but data on bone are scarce. In fact, to our knowledge, only 2 studies have assessed the impact of DR-HC on bone metabolism. The first study [36], analyzing 14 patients with SAI over a 24-month follow-up period, showed an increase in BMD at the lumbar spine and femur neck with DR-HC. The second study [35], evaluating a cohort of PAI patients switching to DR-HC and a parallel unmatched group continuing conventional multiple-daily-dosing therapy over a 72-month timeframe, showed an increase in BMD in the DR-HC group and a decline in the conventional dosing group. Both studies seem to suggest that DR-HC promotes an increase in BMD. However, in the first study, from Frara and colleagues [36], all patients had growth hormone hormone deficiency, and only 2 (14%) were on growth hormone replacement. Moreover, 2 patients (24%) had previous Cushing disease, and data on gonadal status were not available. In our study there were no untreated patients for any hormone deficiency, and patients with previous conditions altering bone health were all excluded, as remission from said diseases could spontaneously improve bone mineralization and architecture and yield unreliable results.

The second study, from Guarnotta and colleagues [35], focused on PAI patients, showing an increase in lumbar spine T-score in the DR-HC group. However, there are several confounding factors in this study as well. First, the authors report that hypovitaminosis D was observed in both groups at baseline and supplemented throughout the study timeframe, with a significant increase in values. Second, hydrocortisone replacement was not stable, and dosages were changed based on clinical signs. Lastly, the comparison between DR-HC and conventional replacement could be biased by the fact that the groups were unmatched; patients on conventional replacement had significantly higher dosages compared to the DR-HC group and to current guidelines, and the switch to DR-HC was based on the presence of comorbidities along with clinical and biochemical signs. On the other hand, patients in our study were originally randomly enrolled in a clinical trial on DR-HC (unbiased selection) and further observed for the present study outcomes, and were treated with stable hydrocortisone regimens, according to current guidelines on GC exposure.

Also, none of the studies has simultaneously evaluated serum markers, BMD, and bone microarchitecture.

Markers of bone metabolism have been used to predict short-term responses to GC administration in endocrine diseases [56, 57] and provide mechanistic insights into the physiopathology of secondary osteoporosis and bone frailty. In our study cohort, levels of ALP and CTX were in the normal range, nor were there any statistical differences between patients with normal and decreased BMD or after the switch to DR-HC. In the study from Guarnotta and colleagues, patients on DR-HC showed a significant increase in OCN and ALP, while the same parameters decreased in the longitudinal conventional therapy group, possibly due to the relatively higher dosages and therapy adjustments [35]. Long-term evaluation and stability of markers of bone resorption in our cohort do not support the hypothesis of a direct effect of DR-HC in increasing bone turnover; neither suggests suppressed bone metabolism at baseline in our patient's cohort. This further confirms the difference between GIO and bone alterations in AI. On the other hand, OCN levels showed a significant increase at 48 months, although the trend was not confirmed at the 72-month time point. Further studies will be required to investigate the effects of a more physiological replacement on osteoblast function.

Subgroup analyses for AI etiology did not show differences in response to DR-HC in all the investigated variables. To our knowledge, this is the first study comparing patients with PAI and SAI, suggesting that SAI patients on adequate replacement for other pituitary deficiencies have similar outcomes in terms of bone metabolism compared to PAI. The comparable results between previous studies on DR-HC, one on SAI [36] and the other one on PAI [35], seem to support this hypothesis. At 72 months, female patients showed a decrease in TBS and intergroup analysis confirmed a significant difference between male and female patients in TBS and a trend toward lower femur neck BMD after adjusting for age and baseline values. Gender-related differences in bone loss have been widely described in current literature, with male patients experiencing a later decline compared to female patients, mostly due to the effects of menopause [58]. Our AI cohort's results seem to align with the general population.

After 6 years, 3 patients progressed from osteopenia to osteoporosis, while no patient with normal bone status developed either. Several patients on conventional replacement in the longitudinal study from Guarnotta and colleagues developed osteopenia or osteoporosis, while no new diagnoses were made in the DR-HC group. This is consistent with our findings and could indicate that patients with baseline impaired bone health might have an increased risk for worsening bone mineralization, despite the switch to a more physiological replacement. Further studies on larger cohorts will help define risk factors stratification for osteoporosis in AI.

In fact, novel biomarkers, such as circadian rhythm disruption and the immune system, are emerging in GC disorders, including AI [3], expanding the definition of adequate replacement from overall daily dosage to more complex evaluations [29]. Accordingly, while relatively low doses of GCs have been suggested to have limited impact on bone quality and quantity [59], the immune alterations (such as an increase in inflammatory immune subpopulations) and metabolic impairment observed under conventional therapy [32] could underlie indirect effects of multiple daily doses GC replacement on bone health.

This study has several limitations: first, given the lack of a control group, our data do not allow a prospective comparison with patients under conventional replacement. Second, not all patients were evaluated at each study time point, even though all patients had at least one longitudinal assessment, and the mean duration of follow-up was close to 5 years. Higher dropout rates are often observed in long-term studies, and due to the stringent exclusion criteria, several patients were excluded due to incident events. On the other hand, the long follow-up duration and thorough evaluation by multiple assessments, along with fewer confounding factors compared to previously published studies, provide a well-rounded analysis and an accurate representation of bone health in PAI and SAI patients.

## Conclusions

Patients with AI showed a high prevalence of impaired bone microarchitecture, while BMD was comparable with the general population of the same age, and replacement GC therapy did not suppress bone turnover in AI. In this study, long-term DR-HC replacement showed no detrimental effects in bone quality and quantity according to BMD, TBS, and bone turnover, potentially limiting the age-related decline in bone mineralization. No differences were observed between PAI and SAI, while sex stratification showed a significant difference in TBS decline between male and female patients. All in all, our results seem to suggest that a more physiological replacement could prevent further decline in BMD, while comparative studies will help elucidate treatment-related effects.

## Data Availability

The dataset generated and analyzed during the current study is not publicly available but is available from the corresponding author at a reasonable request.

## Abbreviations

ACTH: adrenocorticotropic hormone
AI: adrenal insufficiency
ALP: alkaline phosphatase
BMD: bone mineral density
CTX: C-terminal telopeptide
DR-HC: dual-release hydrocortisone
DXA: dual-energy x-ray absorptiometry
GC: glucocorticoid
GIO: glucocorticoid-induced osteoporosis
OCN: osteocalcin
PAI: primary adrenal insufficiency
PTH: parathyroid hormone
SAI: secondary adrenal insufficiency
TBS: trabecular bone score
UFC: urinary free cortisol
